# A Smart System for Remote Monitoring of Patients in Palliative Care (HumanITcare Platform): Mixed Methods Study

**DOI:** 10.2196/45654

**Published:** 2023-05-31

**Authors:** Manuel Ramón Castillo Padrós, Nuria Pastor, Júlia Altarriba Paracolls, Marcelino Mosquera Peña, Denise Pergolizzi, Àngels Salvador Vergès

**Affiliations:** 1 University Hospital Nuestra Señora de la Candelaria Santa Cruz de Tenerife Spain; 2 HumanITcare Barcelona Spain; 3 University Hospital Complex of Ferrol A Coruña Spain; 4 Universitat Internacional de Catalunya, Campus Sant Cugat Barcelona Spain; 5 Innohealth Academy Barcelona Spain

**Keywords:** palliative care, advanced illness, remote monitoring, eHealth, telemedicine, mHealth

## Abstract

**Background:**

Due to the complexities of advanced illnesses and their treatments, it can be difficult for patients in palliative care to maintain their quality of life. Telemedicine interventions in chronic disease management engage patients in their care, provide continuous follow-up by their health care providers, identify symptoms earlier, and allow a quick response to illness-related decline.

**Objective:**

We aimed to detail and reflect on the design of an app and evaluate its feasibility to monitor the clinical situation of patients with advanced illnesses.

**Methods:**

This study used a mixed methods design using qualitative methods to inform app development and design and quantitative methods for data collection and analysis of patient evaluations. Palliative care units in 2 Spanish university hospitals (Nuestra Señora de la Candelaria in Santa Cruz de Tenerife and University Hospital Complex of Ferrol in A Coruña) carried out a literature review, designed the study protocol, and obtained approval from the Ethics Committee from June to December 2020. In addition, focus group meetings were held, and the design and technical development of the app were elaborated on and subsequently presented in the participating palliative care units. From January to March 2021, the app was made public on the App Store and Play Store, and a pilot study with patients was carried out in April to September 2021.

**Results:**

Six focus group meetings were held that included doctors, nurses, app developers, technology consultants, and sponsors. In addition, the technology consultants presented their results 3 times in the participating palliative care units to obtain feedback. After the app’s final design, it was possible to publish it on the usual servers and begin its evaluation in patients (n=60, median age 72 years). Sixty percent (n=36) of the participants were women and 40% (n=24) were men. The most prevalent advanced pathology was cancer (n=46, 76%), followed by other diseases (n=7, 12%) and amyotrophic lateral sclerosis (n=5, 8%). Seventy percent (n=42) of the patients were already in follow-up prior to the start of the study, while 30% (n=18) were included at the start of their follow-up. The information in the app was collected and entered by relatives or caregivers in 60% (n=36) of the cases. The median follow-up was 52 (IQR 14-104) days. In all, 69% (n=41) had a follow-up >30 days (10 were deceased and 9 were missing data). The use of the different sections of the app ranged from 37% (n=22) for the glycemic record to 90% (n=54) for the constipation scale). Patients and caregivers were delighted with its ease of use and usefulness.

**Conclusions:**

Incorporating an intelligent remote patient monitoring system in clinical practice for patients in palliative care can improve access to health services and provide more information to professionals.

## Introduction

Palliative care supports patients with life-threatening illnesses with a focus on disease management and overall quality of life improvement [1]. The need for palliative care is increasing rapidly due to the world’s aging population and the subsequent high prevalence of cancer and other chronic diseases [2]. These illnesses are associated with severe health-related suffering for which eHealth has the potential to intervene. Even in this frail population, the experiences of many have shown that traditional health care can be complemented with initiatives based on eHealth. This broad term refers to applying information and communication technologies (ICT) and networks to manage and deliver health services such as telemedicine and telehealth, mobile health (mHealth), health informatics, and wearable devices. Documented benefits of the adoption of eHealth technologies include more efficient medical consultations, reduced unnecessary displacement, increased accessibility, and the ability to monitor patients’ symptoms, which improves their commitment to care, follow-up, and health-related behaviors [3,4]. Despite the potential interest in integrating ICT initiatives, they have little more than a token presence in many health care administrations. There are four identified types of barriers to the implementation of eHealth: (1) technological (the diversity of existing information systems, (2) organizational (different health care models), (3) human (resistance to change from professionals and patients), and (4) economic (finances and sustainability) [5].

Specific to palliative care, Lundereng et al [6] point out in their recent review that the potential for telehealth to enhance palliative care has been met with several barriers. In addition to the difficulties described in the general population, there are serious concerns about whether telehealth is acceptable in patients with advanced illnesses. The ethical and practical limitations related to their rapid deterioration, advanced age in many cases, physical and cognitive frailty, and generally high needs seem challenging to support with an initiative based on telehealth. Despite these factors, the consulted authors’ synthesis of different telehealth interventions directed at a home-based palliative care population showed many benefits, without additional burdens on patients, and provided valuable information for clinical decision-making. This was echoed in another recent systematic review of the ethical implications and benefits of telemedicine applied to palliative care, which described the positive impact on patient satisfaction and quality of life, although it raised questions related to humanization, as well as ethical implications related to the privacy and security of technology recognized by both patients and health care professionals [7].

The following definition of telemedicine is a starting point: “the provision of medical care at a distance through a variety of telecommunications tools.” This suggests that there are various possible solutions to implement in palliative care. This variety offers the possibility of selecting the most appropriate modality: synchronous or asynchronous; media such as voice, videoconference, and chat; and whether the operating system environment should be computers, mobile phones, or tablets. The main conditions for the success of an initiative related to telemedicine will be whether the project’s design can be adequately implemented with the characteristics and actual needs of patients and professionals. Thus, acceptance will be related to the project’s ability to facilitate clinical management, increase accessibility, improve health outcomes without increasing the burden of care, and provide continuous, high-quality care and follow-up in the location desired by patients and families (usually their homes). Specific to an intervention targeting patients with advanced illnesses, the development team would ideally comprise health personnel members who attend to these patients’ clinical needs alongside programmers with training and expertise in designing and adapting models based on telemedicine. It would also be of great value to obtain feedback on user experience from the patients and their families or caregivers for the optimal final design. However, it is important to note that heterogeneity in the team’s professional background can result in slower project development, as differing expectations and comprehension of clinical needs and strengths or limitations of technologies intersect [6,7].

Here we share the personalization of the HumanITcare platform to the palliative care setting. Emphasis is placed on the developers’ perspectives and workflows for feasibility testing; these results have been reported elsewhere by Castillo-Padrós et al [8]. We believe the considerations and details outlined here will facilitate the implementation of future alternative care initiatives in patients with advanced illnesses and raise awareness among interested clinicians and researchers of the potential of eHealth with this population.

The difficulties in using smart technology by the elderly population have not been the subject of many studies. However, Busch et al [9], in their work on the use of mobile phones (carried out in a cohort of 154 adults aged over 60 years, of whom 45% were older than 70 years), highlight that they used these devices with the general intentions of feeling connected with their social and personal environment, keeping up with the news, and distracting themselves by consulting topics of interest to them on the web. Their families also encouraged the use of these devices, especially for security reasons.

Harris et al [10] followed a cohort of 80 adults with a mean age of 71 years and obtained similar results. The participants described their satisfaction with smart technology. They understood that, although they may have more difficulties than younger people in learning how to use it, the effort was worth it. The most-used device was the mobile phone (75 of 80 participants); this underlay the users’ perception of usefulness. Identified barriers to use included that specific tasks had greater complexity, the cost of the devices, and, in some cases, conflicts related to the loss of privacy.

The design of this study allowed for data collection by the patients themselves or by their relatives or caregivers to ensure that participating in the project was not an inconvenience. Although it was not evaluated in this study due to its design characteristics, it could be interesting to further assess the difficulties that older or dependent patients have with smart technology in future studies.

## Methods

### App Design

**Figure 1:**
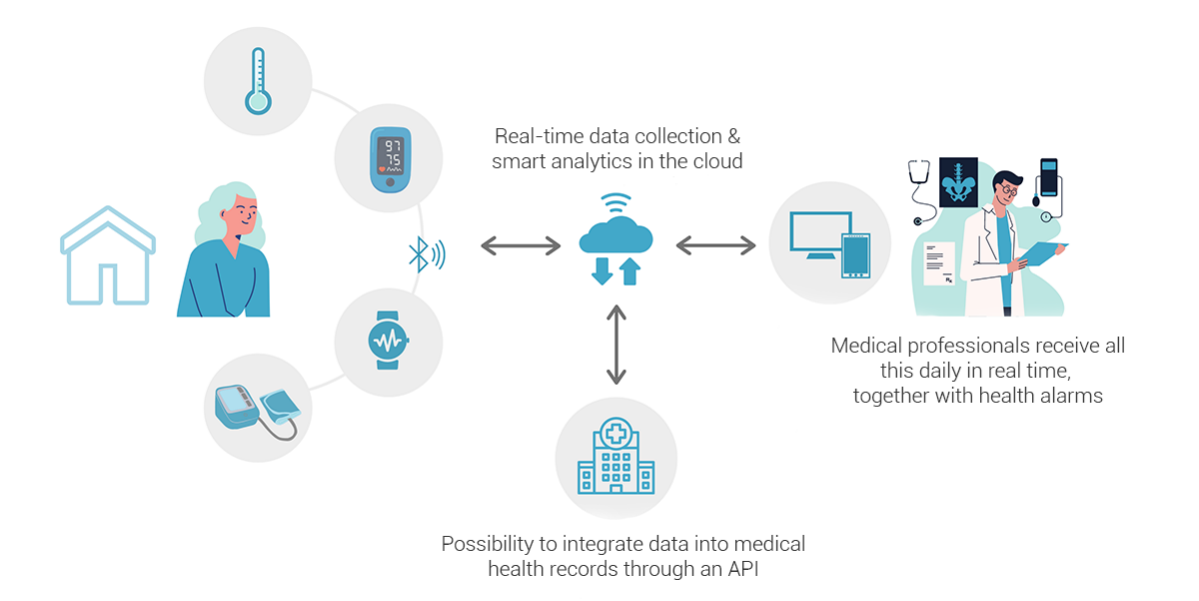
Schematic diagram of the platform. API: application programming interface.

This study is based on the HumanITcare platform. HumanITcare is an intelligent remote patient monitoring system (Figure 1) that can be adapted to different fields of medicine as a framework that connects the patient or caregiver to health professionals with the principal aims of forming treatment plans and follow-up.

### Ethical Considerations

Written informed consent was obtained from each participant after a complete study description. The patients’ data were coded in dissociated databases and could only be accessed by the health care team. The study was approved by the Human Research Ethics Committee of Santa Cruz de Tenerife (2020-217-1) under the Ethical Principles for Medical Research Involving Human Subjects adopted in the Declaration of Helsinki by the World Medical Association (WMA) at the 64th WMA General Assembly in Fortaleza, Brazil, October 2013 and Spanish data protection regulations (Ley Orgánica 3/2018 de Protección de Datos Digitales). Neither the researchers nor the study subjects received financial compensation for their participation.

Each participant in the study was assigned an ID consisting of 8 random characters (eg, d4w192bg), and participant data could only be reviewed using this ID, which ensured data anonymization.

‎Participant data were also encrypted on the access platform for the researchers, technical administrators, and servers. The platform server is in Ireland and complies with the General Data Protection Regulation of May 25, 2018, and the Data Protection Law (Ley Orgánica de Protección de Datos de Carácter Personal, España).

When the patient registered on the platform through the app, the following legal notice regarding data protection appeared on the screen to obtain consent from the patient for data processing:

“This tool does not provide medical advice. It is only intended for information purposes for healthcare professionals. Do not use it as a substitute for professional medical advice, diagnosis, or treatment. Your doctor will review your responses and contact you, if necessary. Confidentiality of your data is important to us. Therefore, we have complied with the established data protection regulations. For more information, please read the detailed legal terms and conditions.”

### Primary Components

This remote monitoring platform has 3 primary components: an app, integrated devices, and a web portal.

#### App for Mobile Phones

The app is available for Android and iOS systems to achieve maximum compatibility. Users register with a secure ID and temporary password. Once registered, the app appears personalized according to the follow-up care plan to which they belong. Depending on the program, users can complete different tasks (eg, recording different measurements and filling in questionnaires from validated scales) to collect data related to their health and well-being. They can consult all this data collected in a personal area available in the app. Communication channels (chat and video calls) are also available to improve accessibility between patients and their health teams.

#### Possibility of Integrating Devices

Wearables and medical devices, such as pulse oximeters, blood pressure monitors, activity trackers, and smart scales, can connect via Bluetooth to the patient’s app to measure vital signs and other medical indicators (eg, physical activity, heart rate, sleep, oxygen saturation, blood pressure, and weight), thus providing an automatic data collection tool. In addition, HumanITcare has different brands of devices integrated into the platform to be device-agnostic and offer the patient the possibility to connect their own devices.

#### Medical Web Portal

Professionals can access a website to manage remote patient monitoring. They can define which (and how often) variables should be monitored depending on each illness and each patient, set alarms when the monitored variables exceed a specific range, choose the connected devices, and customize the questionnaires each participant must answer at a specific time. Once all these parameters have been configured, professionals can view the data through the platform, receive alerts by email when patients report any symptoms or abnormal vital signs from home, communicate with them through the available channels (chat and video calls), and record the medical data of the visits in the patient’s profile.

### App Development

The methodology to adapt the platform to patients in palliative care, as described above, was carried out in January to March 2021 and divided into three stages.

#### Stage 1: Guidance Sessions With Health Personnel

**Figure 2:**
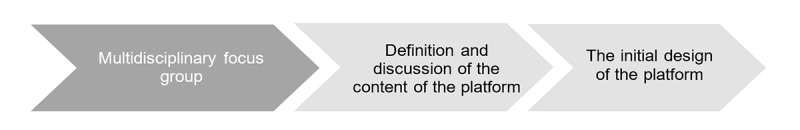
Multidisciplinary focus group.

A total of 6 focus groups were held with palliative care physicians from 2 different hospitals, including methodologists, system developers, and sponsors, with the collaboration of Grupo Ferrer International SA and FollowHealth SL holding multidisciplinary discussions on what should be the best solution for patients in palliative care (Figure 2).

#### Stage 2: Needs Analysis and Technical Requirements

After reviewing the literature and other apps or similar initiatives already developed for other patient populations and considering the results of the discussion groups, a proposal was developed for the app’s content.

**Figure 3:**
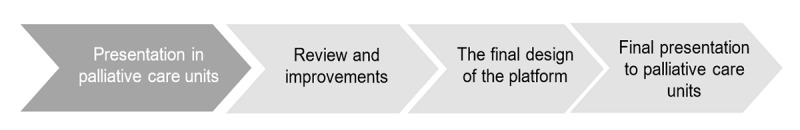
Final presentation to palliative care units.

Once the app’s content was discussed and analyzed, an initial proposal was drawn up, considering ease of use by patients and relatives or caregivers as a central premise for optimal data collection and feedback on variables needed for effective tracking by the population of interest (Figure 3).

Based on the analysis carried out in the first phase, a new app version was created to improve the user experience and functionalities. We selected the following characteristics to determine the health status of patients with advanced illnesses remotely: a registry for clinical variables, a registry for questionnaires, alarms for registered clinical variables, a personal area to review the data of the registered clinical variables, and communication channels, including chat and video calls.

#### Stage 3: Adaptation to Palliative Care and Protocol Approval

**Figure 4:**
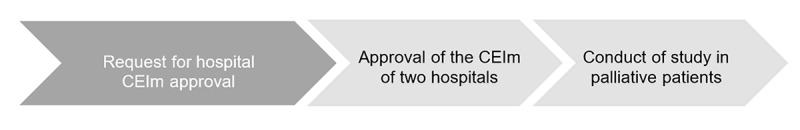
Pilot study at 2 Spanish hospitals. CEIm: Drug Research Ethics Committee.

We sought to adapt the HumanITcare platform specifically to patients in palliative care based on the previously identified requirements and, subsequently, carried out a pilot test among 60 patients from Hospital de la Candelaria (Canary Islands) and Hospital de Ferrol (Galicia). The study was approved by the drug research ethics committees of the hospitals participating in the study (Figure 4).

Most important was that the app be complete and precise, so that professionals could extract the necessary information quickly and easily without adding a burden to their professional duties.

### Final App Design

#### Design for Patients

The content of the app for patients was structured into 4 main sections: clinical measures, questionnaires, a personal area, and chat.

#### Clinical Measures

**Figure 5:**
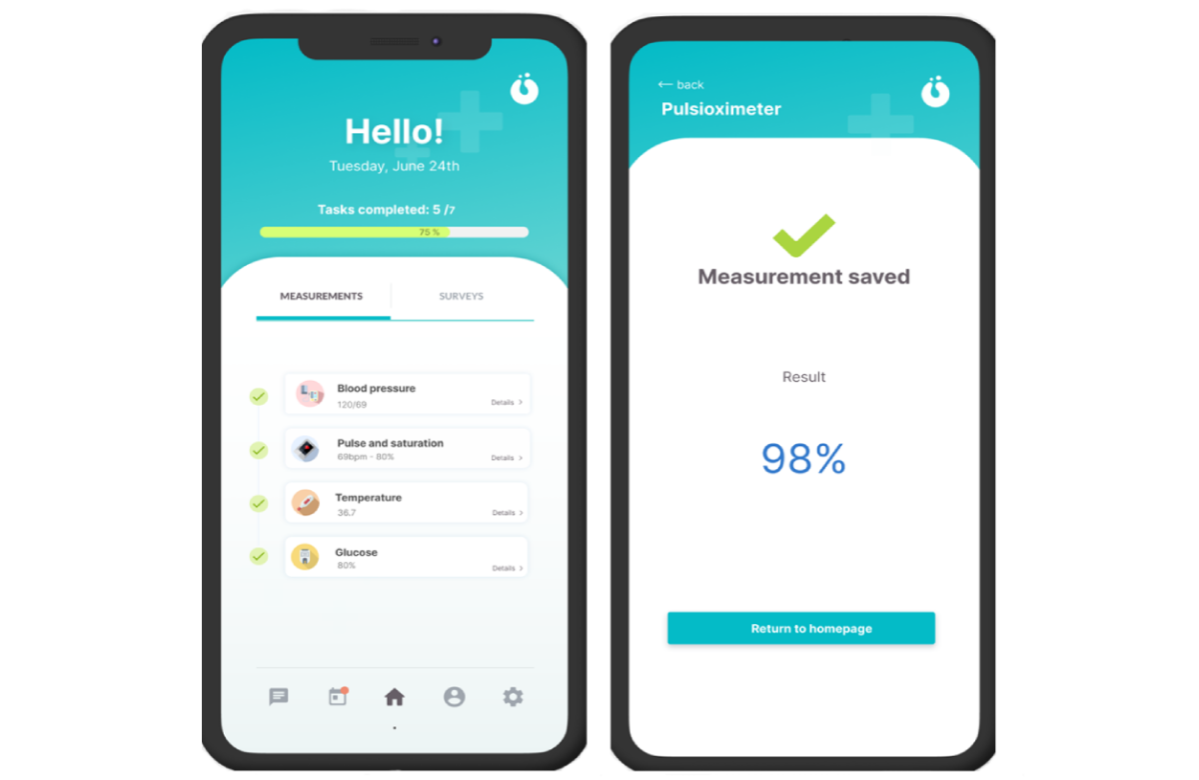
Remote vital sign recording screenshots.

The first section is clinical measures (Figure 5), which includes the following vital signs: heart rate, blood pressure, oxygen saturation, temperature, and glucose. The integrated Bluetooth devices were not used in this pilot. Hence, patients manually reported their vital clinical signs in the app, as it is also an option to register data. Intuitive reminders for the tasks to be carried out were also included for the patients and carers.

#### Questionnaires

**Figure 6:**
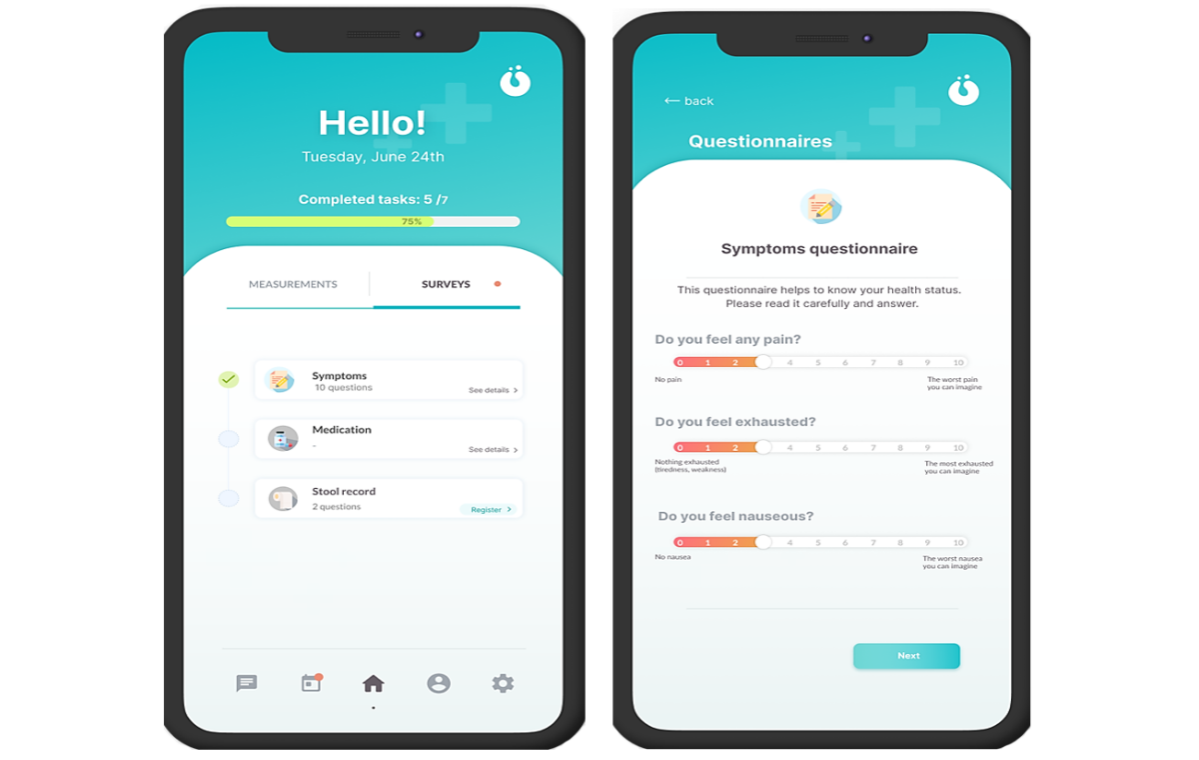
Questionnaire registration.

The second section was questionnaires (Figure 6). Online questionnaires commonly used in clinical practice were used, selected for their particular interest to clinical managers. The questionnaires, and their purposes, were as follows: the revised version of the Edmonton Symptom Assessment System (ESAS), which measures the presence and intensity of the most frequent symptoms experienced by the patient [11]; Rome IV, which analyzes the presence of constipation [12]; the Bristol Stool Scale, which registers fecal shape and type [13]; an adaptation of the Palliative Performance Status (PPS) scale, which provides analysis of the patient’s functional status [14]; and medication adherence, for which items were collected that prompted users to remember the correct intake of opioid medications (“Has the treatment been taken according to the prescription?”; responses were yes or no) and the number of extra “rescue” medications used in the last 24 hours, in addition to those initially prescribed (the reason for this use was also requested, with responses including “treatment of pain,” “respiratory distress,” “anxiety,” and “insomnia”).

#### Personal Area

**Figure 7:**
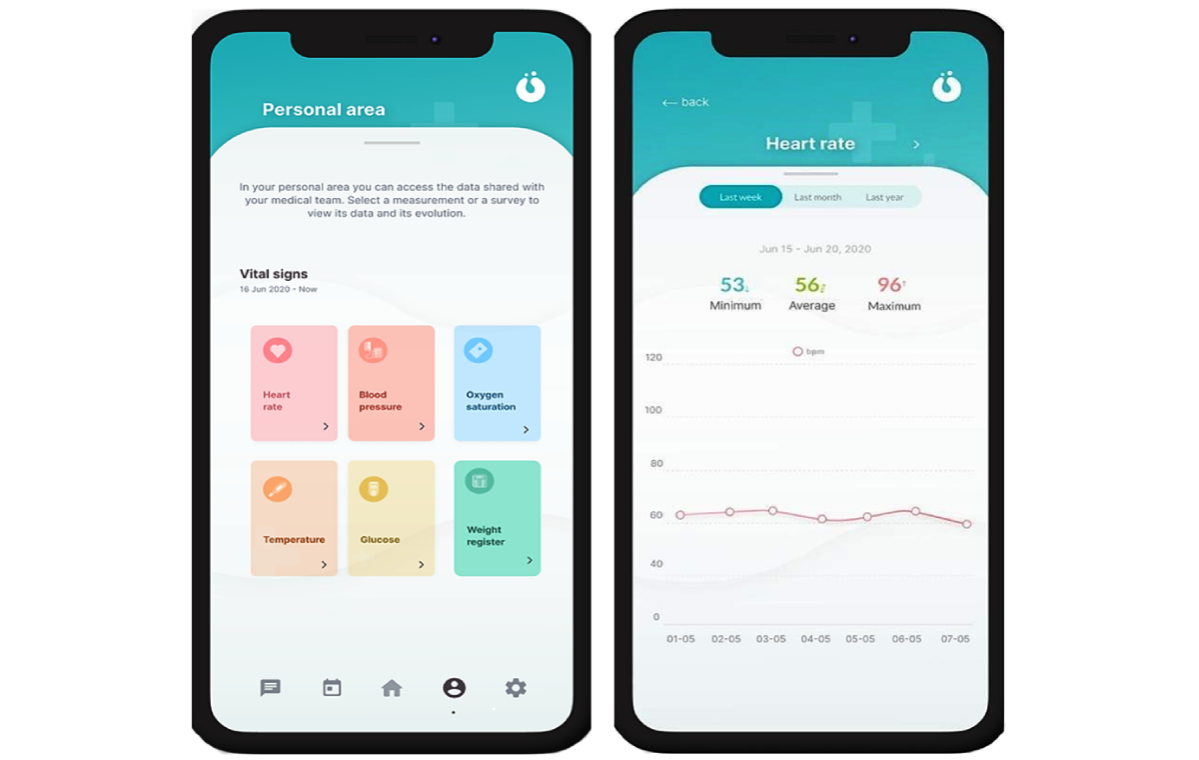
Data diary input by patients.

This digital solution also had a section prepared to display the data entered in the app as a diary, so patients and carers could review the values entered to improve their compliance and support their self-monitoring (Figure 7). Patient information can be displayed on any computer workstation in a web-based format so that professionals can review all the data in real time.

#### Chat

**Figure 8:**
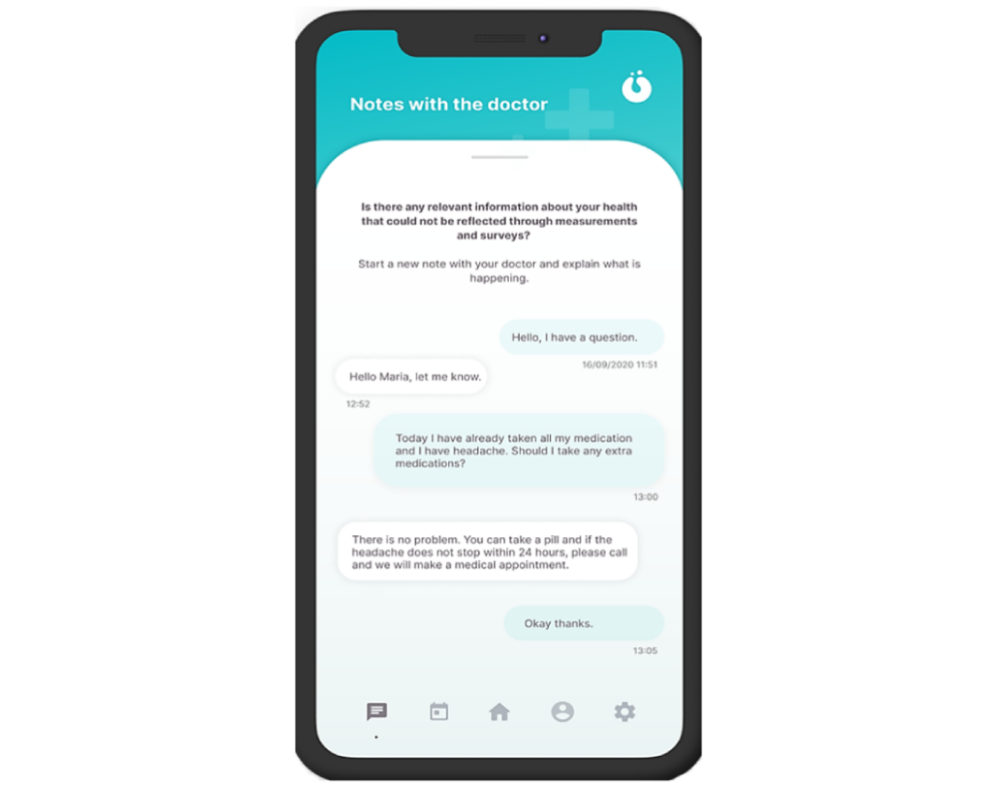
Chat section screenshot.

Patients and professionals can exchange messages about their needs, situation, or clinical process. By downloading the HumanITcare solution, symptoms, vital signs, and medication management can be remotely monitored. In addition, patients, family members, and caregivers could respond to the app’s questionnaires and measurements once a day as they saw fit (Figure 8).

### Design for Professionals

**Figure 9:**
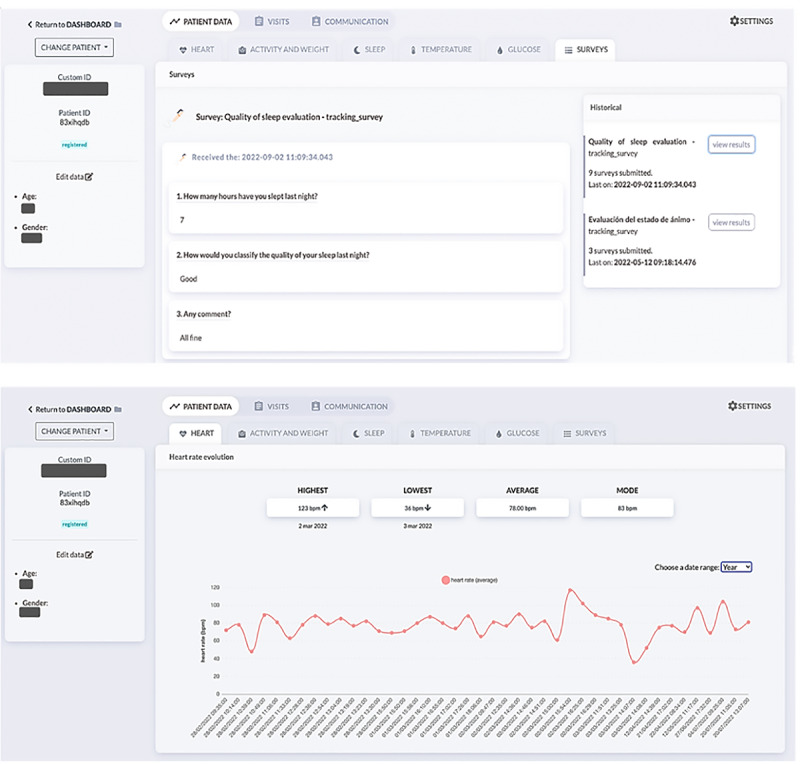
Visualization of professionals’ screens from a workstation using the HumanITcare platform.

The doctors or health care personnel responsible for the patients checked the values entered by the participants daily. They received an alarm if the reference values deviated from normal. In these cases, the patient was contacted as soon as possible by chat or phone (Figure 9).

## Results

### Pilot Testing

After receiving the protocol approval, pilot testing was conducted from April to September 2021 among 60 patients from Hospital de la Candelaria (Canary Islands) and Hospital de Ferrol (Galicia). The following data were collected: demographics, including age, recorded pathology, time of inclusion in the pilot study, and who was responsible for data collection; clinical measurements, including blood pressure, temperature, heart rate, oxygen saturation, and blood glucose; symptom intensity, including monitoring the presence and intensity of the most frequent symptoms (using the revised version of the ESAS); functional status, using an adaptation of the contents of the PPS scale; medication reminders, to assess whether the usual treatment was sufficient for the patient; stools (the presence of constipation was explicitly assessed using an adaptation of the Rome IV criteria); satisfaction questionnaires, including the Post-Study System Usability Questionnaire (PSSUQ); and the use of chat, which provided a new communication channel between professionals and patients or relatives in a secure information environment.

Preliminary results from the data collected have been previously published [8]. The average age of the patients was 72 years. The median follow-up of the patients was 52 days; 69% of the patients completed the 30-day follow-up proposed at the start of the trial.

Table 1 summarizes the characteristics of the recruited patients and the use of the different components of the app.

**Table 1:** Characteristics of the patients (n=60) included in the HumanITcare platform study.

Characteristics		Participants, n (%)
**Gender**		
	Women	36 (60)
	Men	24 (40)
**Advanced pathologies that motivated inclusion in the palliative care program**		
	Cancer	46 (76)
	Respiratory pathologies	7 (12)
	Amyotrophic lateral sclerosis	5 (8)
	Heart conditions	2 (3)
	Renal impairment	1 (1)
**Time point of study inclusion**		
	After follow-up was already started	42 (70)
	At the time follow-up was started	18 (30)
**Responsibility for collecting data in the app**		
	Patients	24 (40)
	Relatives or carers	36 (60)

### Acceptability and Usability

The application’s design is simple, although in all cases, it was ensured that the patient had a family member or caregiver who could help them complete what they could not.

Overall, very positive responses were received, mainly related to the perceived usefulness of the application. One problem mentioned by participants was the length of the PSSUQ and the repetition of items. There was thus a need for feedback, as patients and caregivers only selected items related to overall satisfaction, ease of use, and perceived usefulness. The study focused on acceptance and usability by patients, caregivers, and professionals. However, more research is needed to demonstrate that using the app helps achieve specific care goals, such as improved symptom control and reduced urgent visits.

### Reflections

In order to respond to the needs and deficiencies detected in the clinical care of patients with advanced illnesses, the HumanITcare platform was started with a singular style of shared development.

The HumanITcare solution, characterized by its customization, was adapted to the app based on a review of previous scientific initiatives and the clinical experience of professionals who care for patients with advanced illnesses [8]. Our proposal aimed to detect early changes in health status with the help of mHealth technology to improve medical care for outpatient palliative care and avoid unplanned emergency hospital visits or readmissions. The app and its subsequent study in patients are the results of the coordinated effort of a group of professionals from different fields whose participation in collaborative projects is still exceptional today in our environment. Initially, it involved the union of a team of professionals from 2 public health centers belonging to 2 different health services that are dedicated to the care of highly frail people; research in this field is complex, considering the aforementioned ethical limits, the clinical fluctuations of the patients, and their physical and emotional vulnerability. We had exceptional collaboration from both centers on the part of patients, families, and caregivers, who, in most cases, were very cooperative and welcomed this extended attention and care with genuine enthusiasm.

The technical development for the personalization of the HumanITcare platform was possible due to preexisting familiarity with these applications among the professionals who oversaw it, the response to the needs of the health care workers, and thought about their needs and those of the patients. In addition, having a technical team that continuously monitored the proper functioning of the app and could quickly and effectively resolve doubts and problems raised by patients and professionals contributed significantly to the success of this project.

Most previous initiatives have focused solely on monitoring isolated data, but the HumanITcare platform is comprehensive and consistent. It collects all the data considered especially important for the follow-up of these patients in an intuitive and easy-to-use way for all audiences, thus better controlling each patient’s clinical evolution [6,7]. Until now, data on the correlation between subjective symptom burden and objective parameters have been scarce, so this solution offers an opportunity to collect vital information to fill this gap.

At a qualitative level, the experience and usability data of the patients were very positive. This innovative project aims to complement and help the structures and resources of existing palliative care units to improve effective home care programs that provide comprehensive and coordinated care closely tailored to each patient’s needs.

There are still several steps to take before implementing the systematic integration of this experience in the usual care of this type of patient. Murray et al [15] describe in their review of the key points of the evaluation of telemedicine projects how these interventions must demonstrate their (1) acceptability and usability (Will the target population, ie, patients and health professionals, incorporate and maintain the intervention?), (2) demand (Will it be accepted as necessary by decision-makers?), (3) implementation (Will it maintain its reliability with real-world use?), (4) feasibility (Will it be an acceptable consumption of resources?), (5) adaptation (Can it be used in different contexts without compromising its reliability and integrity?), and (6) integration capacity (Can it be incorporated into existing health resources?). Thus, this initiative and others related to telemedicine face the complexity of valuing projects aimed at transforming systems that are traditionally resistant to change, such as the care field for people with advanced illnesses [16].

## Discussion

### Principal Findings

Here we highlight the HumanITcare platform solution, as it brought together the clinical constants usually evaluated in face-to-face consultations and some of the most-used questionnaires by professionals working with patients with advanced illnesses. The clinical constants are essential for the future development of effective mHealth systems that support remote monitoring of symptoms through wearable devices combined with proactive care for patients at constant risk of rapid or unexpected deterioration of health status. Continuous assessment and critical review of symptoms by patient and physician are the cornerstones of effective outpatient palliative care. Numerous technology-related applications have proven their benefits in engaging patients in their care, promoting treatment adherence, focusing face-to-face assessments on the most critical issues, and improving overall health outcomes.

The results of this project support the conclusions of other studies that patients with advanced illnesses could be the ideal beneficiaries of an intervention focused on telemedicine, and that such intervention can be a helpful complement to their care, so that they can remain in their homes for as long as possible while receiving optimal care [17-19]. The HumanITcare platform, in which the complete, valuable information of patients and caregivers is available via mobile phone with minimal technical problems, seems ideal given that mobile technology has become omnipresent in recent years. Furthermore, in the case of the HumanITcare platform, patients who could not report data due to their physical or cognitive condition could have their caregivers be in charge of data reporting (a task that takes less than 5 minutes); no participant considered this an additional burden.

Even in a population with extreme frailty and high needs, such as patients with advanced illnesses, initiatives based on telemedicine can be successfully developed and enthusiastically accepted [20]. However, the solution must be carefully selected to respond to the real needs of professionals, patients, and those responsible for their care; this is one of the key points to evaluate [21-23]. The results obtained from the development and implementation of the HumanITcare platform encourage us to continue with future studies that evaluate its impact on the desired health outcomes and transfer its use to other areas (for example, home and hospital care teams) with a more significant number of patients. In addition, it was remarkable to see the level of collaboration in the team of clinicians and technicians. Our requirements to consider the objectives of the project as having been achieved include, but are not limited to, (1) gaining necessary knowledge and experience and (2) having a work strategy that allows fluid communication to establish needs, possibilities, and limitations on both sides and offer necessary stability, seriousness, and technical support when working with actual patients in highly complex conditions.

### Lessons Learned

The care and design/technical support teams must be in contact from the beginning of the study to uncover the specific needs of the patients and professionals to be addressed by the app or platform. There must be continuous feedback that quickly incorporates the changes that are considered necessary to resolve the problems that arise. Changes to the overall functions of health care structures and professions are complex, so incorporating these new forms of health monitoring must not add burdens and must show clear benefits. For the incorporation of this app to be sustainable over time, the implemented solution must provide improvements in the health outcomes of patients and, in turn, facilitate the work of professionals. The patients with advanced-stage illnesses and their caregivers or relatives were delighted to participate in this initiative related to new technologies based specifically on mobile phones and handled this technology in a wholly normalized way, so it seems appropriate to continue exploring this line of research and clinical assistance with this population.

### Limitations

This study had limitations. First, although the app was designed to be as straightforward as possible and accessible to anyone, it was introduced as something to be used interchangeably by patients and caregivers. Thus, we could not assess if patients directly experienced problems entering data or with the general use of the app or if they needed a family member or caregiver to assist them in its use. Also, we did not explicitly evaluate, as it was not the aim of the study, whether people with physical or cognitive disabilities or deficits could use the app.

Second, given that the study focused on the acceptability and usability of the app by patients, caregivers, and professionals, the sample size did not provide sufficient power to analyze whether specific care objectives were achieved with the use of the app, such as better symptom control or reduced hospital visits. In line with this, a final limitation was that the pilot testing was only done in 2 hospital units with few patients. However, to recommend its use in other centers or settings (for example, home care units), it would be helpful to demonstrate its role in improving health outcomes. Therefore, future work should aim for a more extensive study with a more significant number of patients across multiple centers.

### Conclusions

The HumanITcare digital solution is proposed as a clinical monitoring tool based on ICT and focused on patients with advanced illnesses. This digital solution optimizes and coordinates activities through intelligent planning, anticipation, and prevention of critical events through its alarm system management. In addition, it automates manual tasks and procedures, facilitates the mutual coordination of medical personnel and patients, carers, and families, and uses a personalized care plan derived from direct monitoring of each patient’s daily signs and symptoms.

Among patients who completed their follow-up study using their mobile phone, more than 80% highlighted the app’s use as an effective form of communication. This app demonstrates that the digital divide is narrowing and that technologies such as those provided by HumanITcare can add value to patient services. Nevertheless, there is some future work to do on this particular platform, including increasing the use of integrated medical devices via Bluetooth to improve usability instead of using manual registration, a function that HumanITcare already has; using voice and image recognition to monitor patients without medical devices at home, using artificial intelligence processes; increasing the use of the video call functionality available on the platform to improve communication between medical professionals and patients from home; and continuing to integrate HumanITcare through an application programming interface with electronic health records (so that the medical team can access all the information on the same management platform) using the Fast Healthcare Interoperability Resource interoperability standard developed by Health Level 7 to enable the electronic exchange of health care data between different systems in the health care industry.

## Abbreviations

ESAS: Edmonton Symptom Assessment System
ICT: information and communication technologies
mHealth: mobile health
PPS: Palliative Performance Status
PSSUQ: Post-Study System Usability Questionnaire
WMA: World Medical Association
